# Scrotal Lymphangiectasia with Penile Elephantiasis in Underlying Lymphatic Filariasis—Challenging the Diagnostic Mind! A Case Report

**DOI:** 10.3390/dermatopathology8010002

**Published:** 2021-01-01

**Authors:** Tejas Vishwanath, Angela Nagpal, Sunil Ghate, Aseem Sharma

**Affiliations:** 1 Department of Dermatology, K.E.M. Hospital, Mumbai 400012, India; tejasvishwanath.igs@gmail.com; 2 DermExpert, Ghatkopar West, Mumbai 400086, India; angie89890@gmail.com; 3 Department of Dermatology, Rajawadi Hospital, Mumbai 400077, India; sdghate_2000@yahoo.com; 4 Dermatology Unit, Skin Saga Centre for Dermatology, Mumbai 400053, India

**Keywords:** filariasis, scrotal lymphangiectasia, penile elephantiasis, dermoscopy, case report

## Abstract

Background: A plethora of diseases manifest as acquired genital lymphangiectasias which clinically manifest as superficial vesicles. They range from infections such as tuberculosis to connective tissue diseases such as scleroderma and even malignancy. Amongst infectious etiologies, lymphatic filariasis leads as the cause for lymphatic obstruction. Despite this, acquired lymphangiectasias due to this cause are not commonly reported. An unusual case of acquired scrotal lymphangiectasia secondary to filariasis is detailed in this paper with dermoscopic and histologic findings. Methods: A 65-year-old male farmer presented with multiple, asymptomatic vesicles over the scrotum with thickened scrotal and penile skin that had occurred for six years. He gave past history of intermittent fever and milky urine, was diagnosed with filariasis and treated with diethylcarbamazine for a year, four years previously. Systemic complaints abated but the peno-scrotal lesions did not. Results: Polarized dermoscopy revealed multiple skin-colored nodules and translucent pale blue lacunae over the scrotum. A few radially arranged linear irregular vessels were noted over the nodules. On histopathology, multiple ectatic lymphatics were noted in the mid and upper dermis with acanthosis and superficial perivascular lymphocytes. Peripheral smear revealed eosinophils; however, microfilariae could not be detected despite repeated diethylcarbamazine provocation and night smears being taken. The findings were compatible with acquired scrotal lymphangiectasia secondary to treated lymphatic filariasis. Local hygiene was advised; however, procedural treatments were refused by the patient. Conclusion: Herein, we report an unusual case of acquired scrotal lymphangiectasia of the scrotum secondary to treated lymphatic filariasis. Very few similar reports exist. To the best of our knowledge, dermoscopic features of this condition have not been elucidated before. This case, detailing an uncommon manifestation of a common disease (filariasis), demonstrates the importance of careful history taking and examination. This was especially so in the present case since only circumstantial evidence of filariasis was noted in investigations. There is a need to heighten awareness of this unusual condition amongst physicians especially if the patient hails from an area endemic for filariasis.

## 1. Introduction

A variety of diseases eventuate in acquired lymphangiectasia, a condition characterized by permanent dilation of cutaneous lymphatics [1]. Diseases that result in acquired lymphangiectasia cause damage to deeper lymphatics, resulting in increased pressure in the superficial lymph vessel which clinically manifests as superficially located vesicles. Varied etiologies are known, encompassing surgery, radiotherapy, malignancies both primary and metastatic, trauma and infections such as tuberculosis [2]. Filariasis, a disease endemic to India, commonly caused by *Wuchereria bancrofti* is characterized by lymphatic obstruction, resulting in a variety of generalized and localized cutaneous manifestations, including over the genitals. Common among these are chylocele, genital elephantiasis and inguinal lymphadenopathy [3]. However, to our knowledge, only three reports detail scrotal lymphangiectasias secondary to filariasis [1,4,5]. We report one such case who presented to a municipality-run public hospital with concomitant dermoscopic and histologic features. Local care, ablative lasers and surgical therapies remain the cornerstone of management.

## 2. Case Report

**Figure 1:**
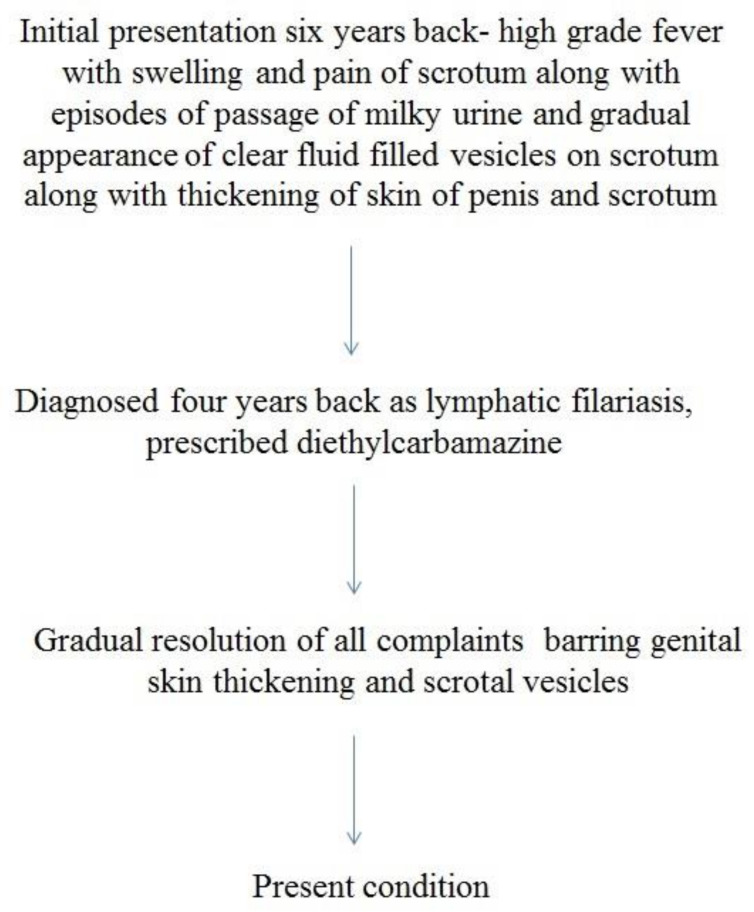
Timeline of patient’s features.

A 65-year-old male farmer presented to a municipality-run academic institute with multiple, asymptomatic, clear fluid-filled lesions of gradual onset on his scrotum and thickening of the skin of the penis and scrotum that had occurred for the past six years. He hailed from the state of Odisha of India and had intermittent episodes of high-grade fever with swelling and pain of his scrotum beginning 6 years previously, along with episodes of the passage of milky urine. A few others in his locality also had similar complaints; however, his family members did not have similar features. He was diagnosed with filariasis four years previously by a local practitioner and had been prescribed diethylcarbamazine (DEC) 100 mg thrice a day for a year, after which his symptoms completely subsided; however, fluid-filled scrotal lesions and thickening of penile skin persisted even after treatment. A detailed history was taken to rule out other common etiologies, especially tuberculosis (absence of weight loss, appetite loss, evening rise in temperature, bladder or bowel complaints, pulmonary symptoms, family history of tuberculosis). A timeline of the patient’s history is shown in Figure 1.

**Figure 2:**
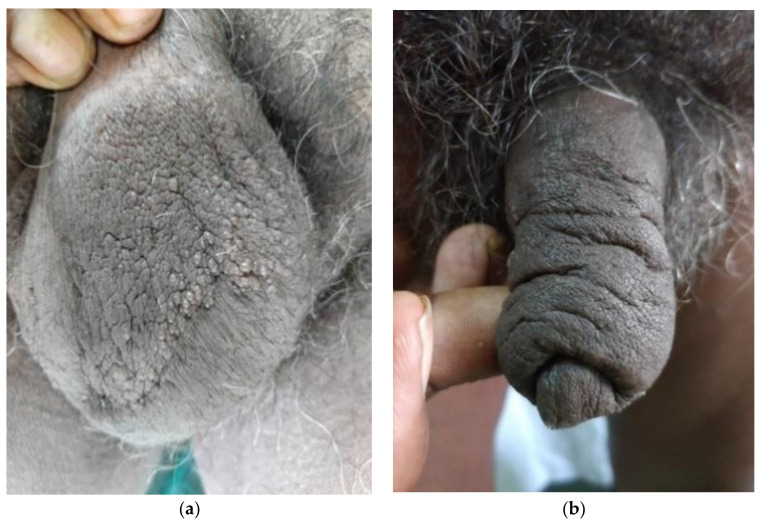
(**a**) Lymphangiectasias on the scrotum with thickened surrounding skin. (**b**) Penile elephantiasis.

**Figure 3:**
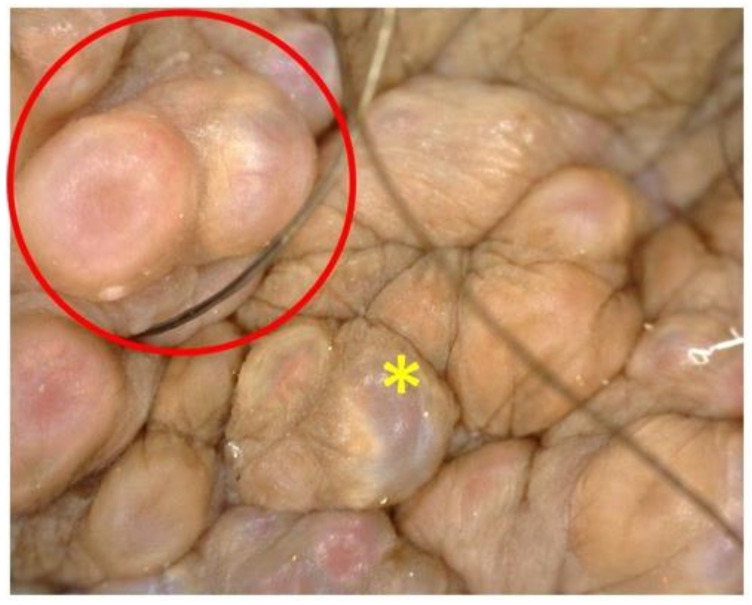
Pale blue lacunae (yellow *) and nodules with radially arranged linear vessels (red circle).

On examination, multiple clear fluid-filled vesicles were noted over the scrotum (shown in Figure 2a). The skin of the penis and scrotum was notably thickened (shown in Figure 2b). The testes were of normal size and consistency but bilateral epididymides, spermatic cords and inguinal lymph nodes were thickened and firm. The rest of the cutaneous and systemic examination was unremarkable. Dermoscopy (Dinolite videodermoscope, polarized mode) revealed multiple, grouped flesh-colored, slightly erythematous nodules on the scrotal skin along with several translucent lacunae with a pale blue hue; some nodules showed radially arranged linear vessels (shown in Figure 3). Rupture of the cysts by sterile needle puncture yielded clear fluid.

**Figure 4:**
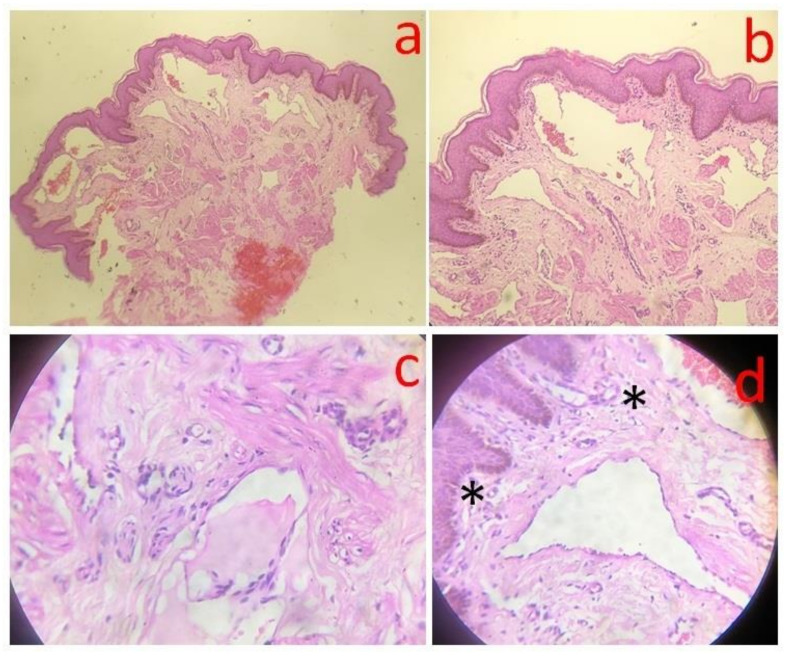
Photomicrograph depicting dilated lymphatic channels in the upper dermis. A valve inside the lymphatic channel is prominent. Acanthosis and superficial perivascular inflammatory infiltrate is also noted (H&E—20×). (**a**) Photomicrograph (H&E stain; 4×), dilated upper dermal lymphatics are prominent. (**b**) At 10× power (H&E stain), mild acanthosis is also noted along with ectatic lymphatic vessels in the papillary dermis. (**c**) Under 40× objective (H&E stain), a valve is clearly noted in the lumen of the dilated lymphatic channel. Dartos muscle is prominent around the latter. (**d**) Under 40× objective (H&E stain), papillary dermal mononuclear infiltrate is discerned along with capillaries (*) and dilated lymphatics.

**Figure 5:**
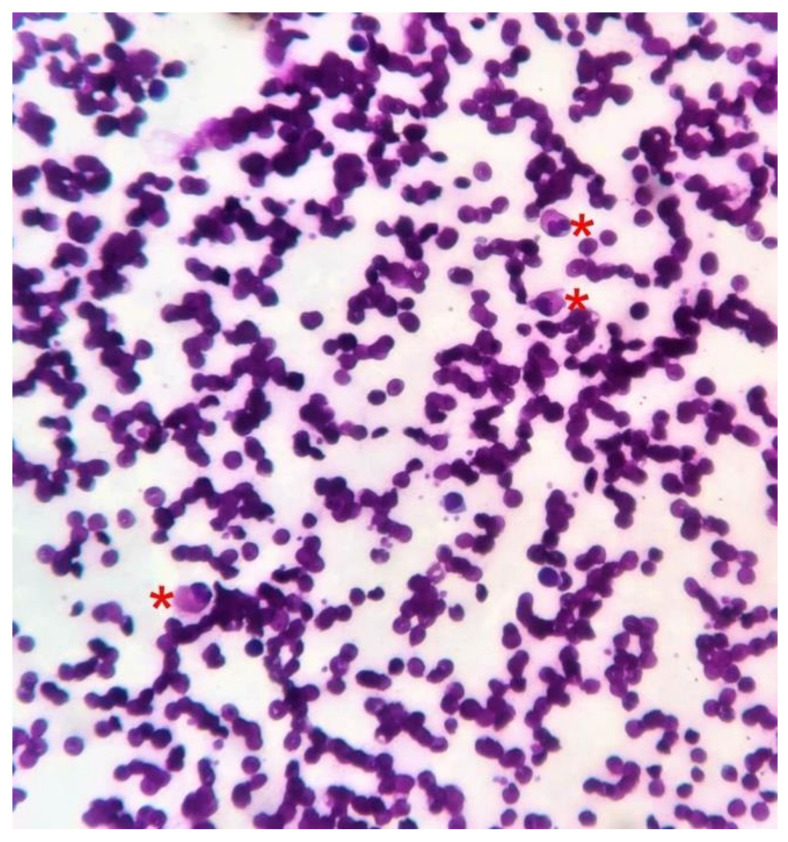
Peripheral smear, multiple eosinophils noted (red *). These suggest a parasitic infestation, in this case, filariasis.

Histopathology of scrotal vesicles (4 mm punch biopsy sample was sent to an ISO-certified private histopathology laboratory) revealed multiple dilated lymphatics along with erythrocytes in the upper and mid-dermis, acanthosis along with mild–moderate superficial perivascular lymphocytes and few dilated blood vessels (hematoxylin and eosin stain, 4×, 10×, 40×; shown in Figure 4). No smooth muscle was noted in the walls of the dilated lymphatics. Repeated examinations of peripheral smears (done in an ISO-certified laboratory at the municipal hospital where the patient presented) from the buffy coat after diethylcarbamazine provocation and night smears (since microfilaremia is noted at night) were negative although multiple eosinophils were noted on the smear (shown in Figure 5). These suggest parasitic antigen stimulation. The differential eosinophil count was 10%, confirming peripheral eosinophilia. Ultrasonography of the inguinoscrotal region revealed an epididymal cyst on the left side with bulky echogenic epididymis and spermatic cords bilaterally. Chest X-ray, abdominal ultrasound, urine and stool examination was normal, discounting the possibility of tuberculosis.

A diagnosis of acquired scrotal lymphangiectasia with penile elephantiasis secondary to treated lymphatic filariasis was kept. Informed consent was sought and recorded from the patient. The patient was counseled about local hygiene and procedural treatments including ablative carbon dioxide laser and surgery. However, he did not consent to procedural treatments and was lost to follow-up.

## 3. Discussion

Cutaneous lymphangiectasia results from the blockage of deeper lymphatics causing increased retrograde pressure and consequent dilatation of dermal lymphatic channels. It may be congenital or acquired. History is vital in distinguishing these entities. Additionally, smooth muscles in the walls of the dilated lymphatics are seen in the congenital variety exclusively [1].

Acquired lymphangiectasias (ALs), as in the present case, manifest as agminate or discrete vesicles or, less commonly, nodules on normal or lymphoedematous skin [1]. A variety of causes result in AL, such as surgery, including radical mastectomy, vasectomy, obstruction of regional lymph nodes due to tuberculosis, radiotherapy, lymphogranuloma venereum, metastasis, scleroderma and Crohn’s disease [6,7,8,9,10].

Lymphatic filariasis is a major public health problem with more than 800 million people in as many as 49 countries remaining under threat of this disease [11]. A raft of genital manifestations are noted which cause considerable social stigma and patient discomfort. Chief among these include genital elephantiasis, inguinal lymphadenopathy, orchitis, lymphangiectasias of the spermatic cord, hydrocele and chylocele, which are caused by lymphatic obstruction [3]. Surprisingly, despite being one of the major diseases causing lymphatic occlusion, very few reports of filariasis-induced scrotal lymphangiectasia are reported [1,4,5]. In the present case, microfilariae were not detected presumably because of the prolonged treatment with DEC the patient had received. The points in favor of our diagnosis were that the patient hailed from a filaria-endemic state (Odisha); history of intermittent fever with chyluria and persistent eosinophilia. A report by Hagiwara et al. describes a similar case of acquired lymphangioma in a long-standing case of filariasis in which microfilaria could not be detected [4]. Russel and Pridie describe another case of a dermal lymphatic cyst secondary to filariasis, in which no microfilariae were detected [5]. They hypothesize that an acquired cause of lymphatic obstruction (such as lymphatic filariasis) unmasks congenital lymphatic deficiency. An important differential diagnosis to be considered is AL secondary to tuberculous scrofuloderma which can present similarly but is managed differently [9]. In endemic areas, thorough workup of the patient needs to be done, as in the present case, to rule out this cause. Other important differential diagnoses include herpes, molluscum contagiosum and verrucae. Eliciting a history of high-risk sexual exposure is of prime importance to rule out these entities. Herpetic vesicles are acute, associated with burning pain and surrounding erythema. Tzanck smear reveals multinucleate epithelial giant cells in the case of herpes. Molluscum contagiosum and verrucae can be easily diagnosed on close examination with a hand lens since the vesicular appearance of lymphangiectasia is absent. Tzanck smear reveals Henderson–Patterson bodies in molluscum contagiosum.

To the best of our knowledge, dermoscopic features of post-filarial scrotal lymphangiectasia have not yet been described. Dermoscopy in the present case revealed varied colors of lacunae and nodules. This could possibly be dependent on epidermal thickness and inflammation: pale blue lacunae sans erythema or vesicles would be expected to have minimal inflammatory infiltrate and normal epidermal thickness, whereas skin-colored or erythematous nodules without a blue hue would exhibit acanthosis overlying inflammatory infiltrate which masks the blue hue. Findings described previously in AL of the vulva and following mastectomy include red and white lacunae and pale septae with irregular, punctiform vessels [10,12]. Dermoscopic features of lymphangioma circumscriptum include yellow, red and blue lacunae (the latter two corresponding to blood) surrounded by pale septae and half and half blister sign due to blood accumulation in the lower part of the vesicle [13]. Of note, septae were not noted in the present case.

Common complications of AL include secondary bacterial infection, emotional and cosmetic discomfort due to the appearance and discharge. Lymphangiosarcoma and squamous cell carcinoma are rare complications [1,14,15].

Treatment includes local care and excision with grafting carbon dioxide laser (especially in vulvar lesions) and local hygiene to prevent secondary bacterial infections [4]. Keloidal tendency must be excluded before procedural treatments.

## 4. Conclusions

Our case documents an unusual finding of scrotal lymphangiectasias in a case of treated filariasis with a novel diagnostic assessment, i.e., dermoscopy. The strengths of the paper include emphasis on the importance of detailed history taking and clinical examination and the meticulous investigations performed to confirm the diagnosis besides ruling out other important differential diagnoses such as tuberculosis and the use of dermoscopy, a new diagnostic aid which gives a new perspective.

The limitations include a single case, the signs of which would need reproduction and validation with subsequent cases.

## Data Availability

The data presented in this study are available on request from the corresponding author. The data are not publicly available due to patient confidentiality and absence of electronic medical record (EMR) system.
